# Artificial autonomy and algorithmic paternalism: AI shaping human autonomy and decision-making

**DOI:** 10.3389/frai.2026.1860239

**Published:** 2026-06-26

**Authors:** Bjørn Hofmann

**Affiliations:** 1 Centre of Medical Ethics, The University of Oslo, Oslo, Norway; 2 Department of Health Sciences, The Norwegian University of Science and Technology (NTNU), Gjøvik, Norway

**Keywords:** accountability, agency, artificial intelligence, authenticity, autonomy, bias, ethics, integrity

## Abstract

While artificial intelligence (AI) systems can enhance human autonomy, they also challenge human self-determination as they exhibit “artificial autonomy” and enact “algorithmic paternalism”. This article examines how AI can influence three core preconditions of autonomy — understanding, decision-making competence, and voluntariness. Moreover, it analyses breaches of three standard conceptions of autonomy (as agency, as authenticity, and as relational autonomy). The article further describes how the agentic appearance and perceived authority of AI systems can undermine human autonomy and enact “algorithmic paternalism” corresponding to traditional forms of paternalism. The complexity and opacity of AI systems challenge human autonomy in special and unprecedented ways which may have vast implications for individuals and societies. Therefore, identifying artificial autonomy and algorithmic paternalism and their mechanisms is crucial for preserving human autonomy.

## Graphical abstract

**Figure:**
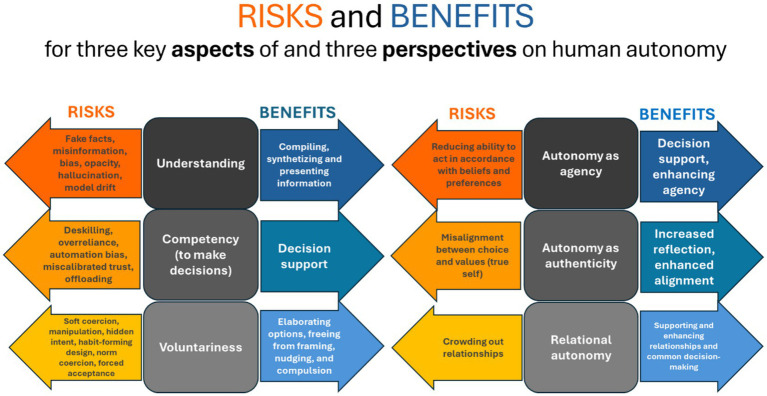

## Introduction

Artificial intelligence (AI) systems are widely regarded as disruptive technologies with extraordinary potential, envisioned to vastly improve a wide range of aspects of human life. It is envisaged to increase effectiveness, innovation, accuracy, decision-making, personalization, cost savings, health, safety, and security. At the same time, they raise profound technical, epistemic, ethical, and legal issues. ‘Hallucination’ (fabrication of output) (Alkaissi and McFarlane, 2023), bias (systematic error) (Hofmann, 2025), ‘model drift’ (deterioration over time), incomprehensibility (the “black box problem”) (London, 2019; Hassija et al., 2024), deskilling (Budzyń et al., 2025; Ferdman, 2025; Green, 2019; Natali et al., 2025), and lack of alignment with human values are only some of the identified challenges that threaten to undermine the benefits of and trust in AI/ML.

As the AI systems are predicted strongly to influence human decision-making, there is a risk that AI may undermine human autonomy and become paternalistic. Hence, while being envisioned to enhance human autonomy, e.g., by extending our choice architecture, clarifying our preference structure, and informing and supporting our decision-making capacity (Khosravi et al., 2024; Puttaraju, 2023), AI systems can also undermine human self-determination and agency (Lu, 2024; Gstrein, 2024; Koralus, 2025). For example, when their outputs are inaccurate, fake, biased, unstable, or incomprehensible, they can undermine understanding, and thereby human autonomy. Moreover, AI systems can direct attention, define goals (Subías-Beltrán et al., 2025), and sway decisions (Subías-Beltrán et al., 2025). They can also result in uncritical trust, automaton bias, and overreliance on AI in decision-making (Lutz et al., 2025; Gesnot, 2025; Olteanu et al., 2025). In particular, AI systems may induce manipulation, adaptive preference formation, deception and adaptive belief formation, as well as loss of competency, options, and freedom (Prunkl, 2022). All these factors may undermine human autonomy and introduce various kinds of algorithmic paternalism.

Autonomous AI systems have become pervasive (Ajayi et al., 2024), and have come to act as (implicit and explicit) moral agents and moral proxies, solving moral problems and making “autonomous decisions on behalf of human stakeholders” (Bonnefon et al., 2024). Moreover, Embodied AI (EmAI) have given AI systems “a physical ‘body’, enabling direct interaction with the world” (Liu et al., 2025). Thus, AI systems have become autonomous agents interacting with and influencing human agency and autonomy. Accordingly, AI systems “directly influence individuals’ ability to exercise free and informed choice” (Subías-Beltrán et al., 2025). Being directed by or relying on algorithmic recommendations may result in ceding control over decisions to an artificial entity with all the hallmarks of traditional paternalism.

Accordingly, the objective of this article is to investigate how human autonomy is influenced and undermined by AI systems and how this trigger existing and special kinds of paternalism. This will be done by scrutinizing two specific phenomena:

1. Artificial autonomy, i.e., how human autonomy is modified (enhanced or diminished) by AI systems.
2. Algorithmic paternalism, i.e., how AI systems can enact existing and special forms of paternalism.

## Methods

To investigate this, the study first conducted an unsystematic review to provide examples of how autonomy and agency is mentioned in key documents in AI ethics. Then it performed a basic thematic content analysis (Cavanagh, 1997; Hsieh and Shannon, 2005) of seminal publications on how AI systems can promote and undermine autonomy. To do so it applied a standard conception of autonomy from normative ethics (Gracia, 2012; Oshana, 2006), i.e., autonomy defined in terms of understanding, decision-making competence, and voluntariness. The articles were searched for on GoogleScholar and the selection criteria were (1) clarity of the example or argument, (2) originality, i.e., whether the article provided a new example or argument, (3) relevance, and (4) whether the article was referred to by others. As the aim was to find examples and arguments, and not being exhaustive, the search was stopped when examples or arguments were repeatedly encountered. Then the reference best satisfying the criteria was chosen.

Thereafter, the study conducted a basic thematic content analysis (Cavanagh, 1997; Hsieh and Shannon, 2005) of seminal publications on how AI systems can promote and undermine and standard conceptions of autonomy in normative ethics (agency, authenticity, and relational autonomy). Again, the articles were searched for on GoogleScholar and the selection criteria were the same as listed above. The aim here was to find examples and arguments, and not to be exhaustive. Therefore, the search was stopped when examples or arguments were repeatedly encountered. Then the reference best satisfying the criteria was chosen.

Lastly, the study conducted a search for seminal publications on “algorithmic paternalism” to investigate how this influences human autonomy. Again, the articles were searched for on GoogleScholar, with the same selection criteria as listed above. Also here, the aim was to find examples and arguments, and not to be exhaustive. The search was therefore stopped when examples or arguments were repeatedly encountered. Then the reference best satisfying the criteria was chosen. The findings were analysed according to standard conceptions of paternalism in normative ethics (weak/strong, soft/hard, and libertarian) (Dworkin, 2020).

## Results

### Artificial autonomy

The autonomous potential of AI systems is explicitly mentioned in the definition in the European AI act: “‘AI system’ means a machine-based system that is designed to operate with varying levels of autonomy and that may exhibit adaptiveness after deployment, and that, for explicit or implicit objectives, infers, from the input it receives, how to generate outputs such as predictions, content, recommendations, or decisions that can influence physical or virtual environments” (Smuha, 2025). The challenge of respecting autonomy is extensively mentioned in the AI Ethics literature (Karimian et al., 2022; Morley et al., 2020; Morley and Floridi, 2024; Khan et al., 2022; Mittelstadt et al., 2016; Al-Hwsali et al., 2023; Benzinger et al., 2023). As explicitly stated by Floridi and colleagues “when we adopt AI and its smart agency, we willingly cede some of our decision-making power to machines” (Floridi et al., 2018).

Table 1 provides an overview of where and how autonomy is mentioned in a selection of key documents. While it has been pointed out that “autonomy has received significantly less attention from the scientific community than other prevalent themes, such as ‘fairness’ or ‘explainability’” (Prunkl, 2022), the topic deservedly is gaining traction (Ajayi et al., 2024, Annoni, 2025, Berber and Mijić, 2026, Buijsman et al., 2025, Cooper, 2025, Del Valle and Lara, 2024, Regulation (EU) 2024/1689 laying down harmonised rules on artificial intelligence (Artificial Intelligence Act), 2024, Krook, 2025, Laitinen and Sahlgren, 2021, Liu et al., 2025, Marchegiani, 2025, Mhlambi and Tiribelli, 2023, Salikutluk et al., 2025, Sheintul, 2025, Vaassen, 2022).

**Table 1:** Autonomy and agency mentioned in key documents in AI ethics (author’s emphasis).

Framework	How autonomy is addressed (directly or indirectly)
AI4People (Floridi et al., 2018)	“Humans should always retain the power to decide which decisions to take, exercising the freedom to choose where necessary, and ceding it in cases where overriding reasons, such as efficacy, may outweigh the loss of control over decision-making”
Global landscape (Jobin et al., 2019)	“Freedom and autonomy are believed to be promoted through transparency and predictable AI, by not “reducing options for and knowledge of citizens,” by actively increasing people’s knowledge about AI, giving notice and consent or, conversely, by actively refraining from collecting and spreading data in absence of informed consent.”
IEEE Ethically Aligned Design (EAD) (IEEE Standards Association, 2018)	AI systems “are objects as all tools typically were, but simultaneously, as they are endowed with a capacity for agency, they are also subjects.”
The European Commission’s High-Level Expert Group on AI (Pekka et al., 2018)	‘Respect for autonomy’ is the first of four key ethical principles of the European Commission’s High-Level Expert Group’s Ethics Guidelines for Trustworthy AI. Moreover, the Group presents seven “key requirements” that include human agency and oversight
European Group on Ethics in Science and New Technologies (2018)	*Statement on Artificial Intelligence, Robotics and ‘Autonomous’ Systems:* Autonomous systems “must not impair [the] freedom of human beings to set their own standards and norms and be able to live according to them”
OECD Recommendation of the Council on Artificial Intelligence (Organisation For Economic Co-Operation And Development, 2019)	“AI actors should respect the rule of law, human rights, democratic and human-centred values throughout the AI system lifecycle. These include non-discrimination and equality, freedom, dignity, autonomy of individuals, privacy and data protection, diversity, fairness, social justice, and internationally recognized labour rights. This also includes addressing misinformation and disinformation amplified by AI, while respecting freedom of expression and other rights and freedoms protected by applicable international law.”
The Asilomar AI Principles (Future of Life Institute, 2017)	“Highly autonomous AI systems should be designed so that their goals and behaviors can be assured to align with human values throughout their operation.”
Montréal Declaration for Responsible Development of Artificial Intelligence (Abrassart et al., 2018)	“the development of AI should promote the autonomy of all human beings and control … the autonomy of computer systems”. Autonomy is the second principle of the declaration: “AIS must allow individuals to fulfil their own moral objectives and their conception of a life worth living.”
ITI AI Policy Principles (Council., I. T. I, 2017)	“Consider including provisions within legislation that are intended to provide users with sufficient information to understand decisions of an AI system that may negatively affect their fundamental rights and provide users with the ability to review and/or challenge such decisions.”
Microsoft AI principles (2025) (Council., I. T. I, 2017)	Through prerequisites for autonomy: “AI systems should be understandable.” “AI systems should be secure and respect privacy.”
the European Commission’s White Paper on Artificial Intelligence (European Comission, 2020)	“Human oversight helps ensuring that an AI system does not undermine human autonomy or cause other adverse effects. The objective of trustworthy, ethical and human-centric AI can only be achieved by ensuring an appropriate involvement by human beings in relation to high-risk AI applications”

While human autonomy is addressed in a wide range of documents on ethics, it is not always addressed explicitly, and in some key documents it is not addressed at all, e.g., in the OpenAI Charter (OpenAI, 2018).

Autonomy is a key concept in Western liberal democracies, underscored by legislation founded in basic beliefs in individual freedom, self-determination, and the rights of individuals to make their own decisions. “Autonomous choice is a cornerstone of our social, economic, psychological and political systems” (Gal, 2018). It shapes democratic governance by promoting the protection of civil liberties, informed consent, and the capacity for individuals to participate actively in decision-making processes. In legislation and regulation, human autonomy informs policies that respect personal agency while balancing societal interests, thus ensuring that individual rights are upheld in a framework of moral and ethical responsibility. Ultimately, human autonomy is seen as essential for fostering a just society where individuals are empowered to define their own lives and contribute meaningfully to civic discourse.

Hence, if AI systems undermine autonomy, it undercuts a foundation for liberal democracies.

### Undermining basic aspects of autonomy

While autonomy can be defined in very many ways (Brock, 1988; Christman, 2003; Dworkin, 1988; Feinberg, 1982; May, 1994; O'neill, O., 2002; Pettigrew, 2021; Scoccia, 1990; Faden and Beauchamp, 1986; Kant, 2005; Mill, 1998), there is fair agreement on three main preconditions for human autonomy, i.e., understanding, competency, and voluntariness (Gracia, 2012; Oshana, 2006). AI systems can influence these preconditions in several ways.

On the one hand, AI systems clearly can increase *understanding* by compiling, synthetizing and presenting information. On the other hand, they can instigate false beliefs due to bias, hallucination, and model drift and decrease understanding by opacity and lack of explainability. Producing fake facts, misinformation, and framing defaults. There is a rich literature and many examples of how AI systems can enhance and undermine understanding (Ai, 2025; Xie et al., 2026) and thereby affect autonomy.

Correspondingly, AI systems can influence human decision-making competence^1^. While AI/ML-based decision-support systems can facilitate improved decision-making, AI systems may also deteriorate decision-making competence. Deskilling is but one example, as repeatedly delegating tasks to AI reduces opportunities to practice relevant skills, to reflect, and to deliberate (Ferdman, 2025; Berzin and Topol, 2025). For example, endoscopists have demonstrated deskilling in cancer assessment and deliberation after exposure to artificial intelligence in colonoscopy (Budzyń et al., 2025).

Overreliance and automation bias (Lutz et al., 2025; Gesnot, 2025; Olteanu et al., 2025) is another threat to competency as users may come to accept AI outputs too readily, ceasing to apply critical checks, weakening judgment and making users less able of detecting errors and less creative in deliberation (Romeo and Conti, 2025). For example a recent systematic review has shown that students prefer efficient cognitive shortcuts, even amidst the ethical issues presented by AI technologies (Zhai et al., 2024). The same challenge occurs for mis-calibrated trust (wrong confidence assessment) (Mehrotra et al., 2024) and cognitive offloading, which may instigate superficial acceptance of outputs without deliberation (Hooper, 2025). Yet another challenge to human autonomy by undermining decision-making competence is AI systems’ influence on people’s choice architecture (13) (Duane et al., 2025). Additionally, there are “concerns about the Second Singularity - a scenario in which decision-making autonomy is increasingly ceded to AI, weakening human oversight” (Natali et al., 2025).

Correspondingly, AI systems may influence autonomy by enhancing or undermining human *voluntariness*. On the positive side, such systems may augment voluntariness by providing and informing about more options and by revealing and freeing from framing, nudging, or compulsion. On the other hand, AI systems may provide covert nudging (Beraldo, 2025; Calboli and Engelen, 2025; Duane et al., 2025; Fritzen, 2023; Sheintul, 2025; Shin, 2025), for example by soft coercion (Shin, 2025), tailoring choice architecture (order, defaults, emphasis) in ways the user does not notice, and nudging decisions without the person’s conscious endorsement. As pointed out by Karen Yeung, “Big Data driven nudging is … nimble, unobtrusive and highly potent, providing the data subject with a highly personalized choice environment - hence I refer to these techniques as ‘hypernudge’” (Yeung, 2019).

Correspondingly, AI systems can exploit personal vulnerabilities, for example by using intimate profiling (of affections, fears, tendencies) to target psychological weaknesses, driving decisions the person would otherwise not use. Feeding information and ads on luxury products to insecure persons anxious about social status, is but one example. Relatedly, AI systems can generate compulsion by a habit-forming design, for example by making people act out of habit or craving rather than deliberate choice.

Hidden intent in terms of deceptive or opaque persuasion, is another way that AI systems may undermine voluntariness (and understanding). Promoting political messages disguised as peer grassroot content is but one example how AI systems can make recipients endorse a position believing it reflects peer opinion rather than targeted persuasion. Other mechanisms are the erosion of meaningful alternatives (limiting perceived options), social pressure amplified by algorithmic visibility (norm coercion), and economic or infrastructural dependency (forced acceptance). An example of the latter is a municipality’s AI scheduling system that is the only way to access public benefits, and where residents must accept automated conditions they find objectionable if they want services (Polcumpally, 2025).

**Figure 1:**
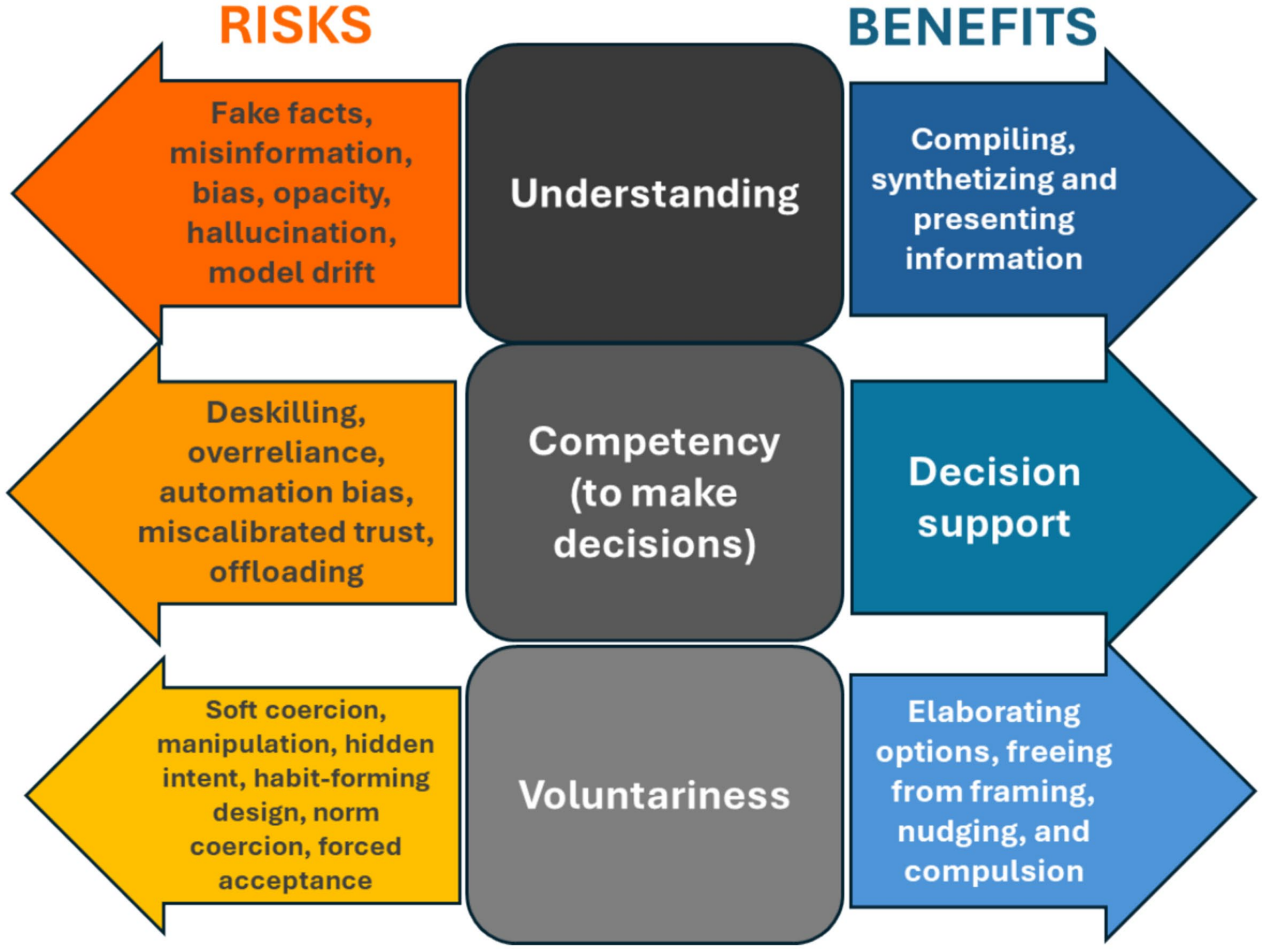
Summary of how AI systems can promote and undermine autonomy, i.e., through understanding, decision-making competence, and voluntariness.

This indicates that while AI systems may augment autonomy by enhancing understanding, decision-making competence, and voluntariness, they may also do the opposite by a wide range of mechanisms summarized in Figure 1.

The various threats to undermining autonomy indicate specific risks that work as red flags and that point to strategies to avoid artificial autonomy. For example, there are ways to measure and mitigate biases (Hofmann, 2025; Chen et al., 2024; Gray et al., 2024), deskilling (Berzin and Topol, 2025; Natali et al., 2025), and malicious nudging (Mills, 2024). Outlining such specific measures and strategies is the next step, which warrants separate publications. Here I try only to lay the foundation for such future work.

### AI challenging three conceptions of autonomy

Beyond shaping the three constitutive requirements for human autonomy, AI systems may influence autonomy differently depending on the conception of autonomy. While notoriously complex, the concept of autonomy is generally understood to mean an individual’s effective capacity for self-governance (Young, 2017; Koralus, 2025). This implies that persons can act based on beliefs, values, motivations, and reasons that are genuinely their own (3,25). Three basic conceptions are autonomy as *agency*, as *authenticity*, and *relational autonomy*.

According to the first, individuals can act on their beliefs, values, and reasons, requiring decision-making capacity, independence, self-direction, and responsibility. The second conception emphasizes individuals’ alignment of their choices, beliefs, and values with their true selves. It requires that persons can express their thoughts, beliefs, and choices; that their choices are aligned with their values; and that they are able to reflect on desires, motivations, and implications of their choices. Additionally, *relational autonomy* emphasizes that persons’ capacity for self-governance and decision-making is significantly influenced by their relationships, social interactions, and the broader cultural and institutional frameworks in which they exist.

AI systems may challenge all these conceptions of autonomy.

In terms of *loss of agency*, persons can become disempowered by AI systems as human skills and decision-making capacities will be outcompeted (Krook, 2025). Moreover, humans may also become overwhelmed by AI systems, which may disrupt their choice architecture and introduce a kind of AI-nudging that undermines autonomy (Koralus, 2025). In particular, AI systems may have a range of effects on users’ choices, e.g., in the choice of directive algorithms, in defining decision parameters, in influencing biases, in nudging strategies, and in altering the number and quality of choices (Gal, 2018). For example, hospital scheduling algorithms maximizing throughput may come to prioritize short visits and automatic discharge, pressuring clinicians to cut counseling time and reducing patients’ ability to participate in shared decision making.

AI systems may also use predictive analytics to foresee individuals’ needs and preferences, pre-emptively offering choices and simulate outcomes for different choices, providing individuals with insights into potential consequences, which may influence their agency in decision-making process. Agency can also be reduced by harm avoidance: “When AI systems are calibrated to avoid harm at all costs, they implicitly deny the moral legitimacy of voluntary risk-taking. They reduce moral agency to compliance, and innovation to liability mitigation. Such design logic is isomorphic with the worst tendencies of the administrative state: bureaucratic, collectivist, and depersonalizing” (Wright, 2025). Another source of loss of agency is given by the attribution of agency to AI systems. AI systems and robots are increasingly given names and “synthetic authority” (Startari, 2025) and attributed personality, which directly may affect the agency of persons using (or being used or manipulated by) such systems (Legaspi et al., 2019). Hence, AI systems may reduce human autonomy by reducing their agency. See more on loss of agency and autonomy below.

Correspondingly, AI systems may challenge *authenticity* in that they may distort the alignment between individuals’ beliefs, values, and choice, e.g., because they reduce their opportunity or reflections on their motivations. For example, AI systems may evoke and strengthen a person’s impulses and reducing the person’s time or capacity for reflection, as it happens in social media’s attention capture. While AI systems may increase preference awareness and encourage preference and choice reflection, they can also bias and manipulate such considerations, e.g., through habit formation, framing, defaults, and forming choice architecture. Hence, by influencing or directing second order preferences or beliefs about the relationship between first and second order preferences, or by hampering the influence of second order preferences on first order preferences, AI systems may undermine authenticity.

Lastly, AI systems may also interfere with *relational autonomy* both in terms of crowding out relationships (as they may seem less relevant for decision-making) or because the systems themselves may represent strong relationships. For example, para-social AI companions may replace human social interactions or shaping persons’ will-formation. While AI algorithms can analyse social interactions and power dynamics, and provide personalized recommendations, they may also come to reduce interpersonal skills, impose norms and defaults, marginalize voices, and enhance power imbalances. However, it has been argued that a relational conception of autonomy can better address the potential harms of AI systems than rational conceptions (Mhlambi and Tiribelli, 2023).

Correspondingly, AI systems may introduce a new type of nudging, undermining autonomy. While nudging has been viewed as a compromise between libertarianism and paternalism, AI-based nudging may undermine this balance, as it becomes a “subtle but sweeping form of digital rhetoric, one that can invisibly shape the collective mind, inhibit the decentralized adaptive learning that underlies human truth-seeking, and ultimately undermine human autonomy” (Koralus, 2025).

**Figure 2:**
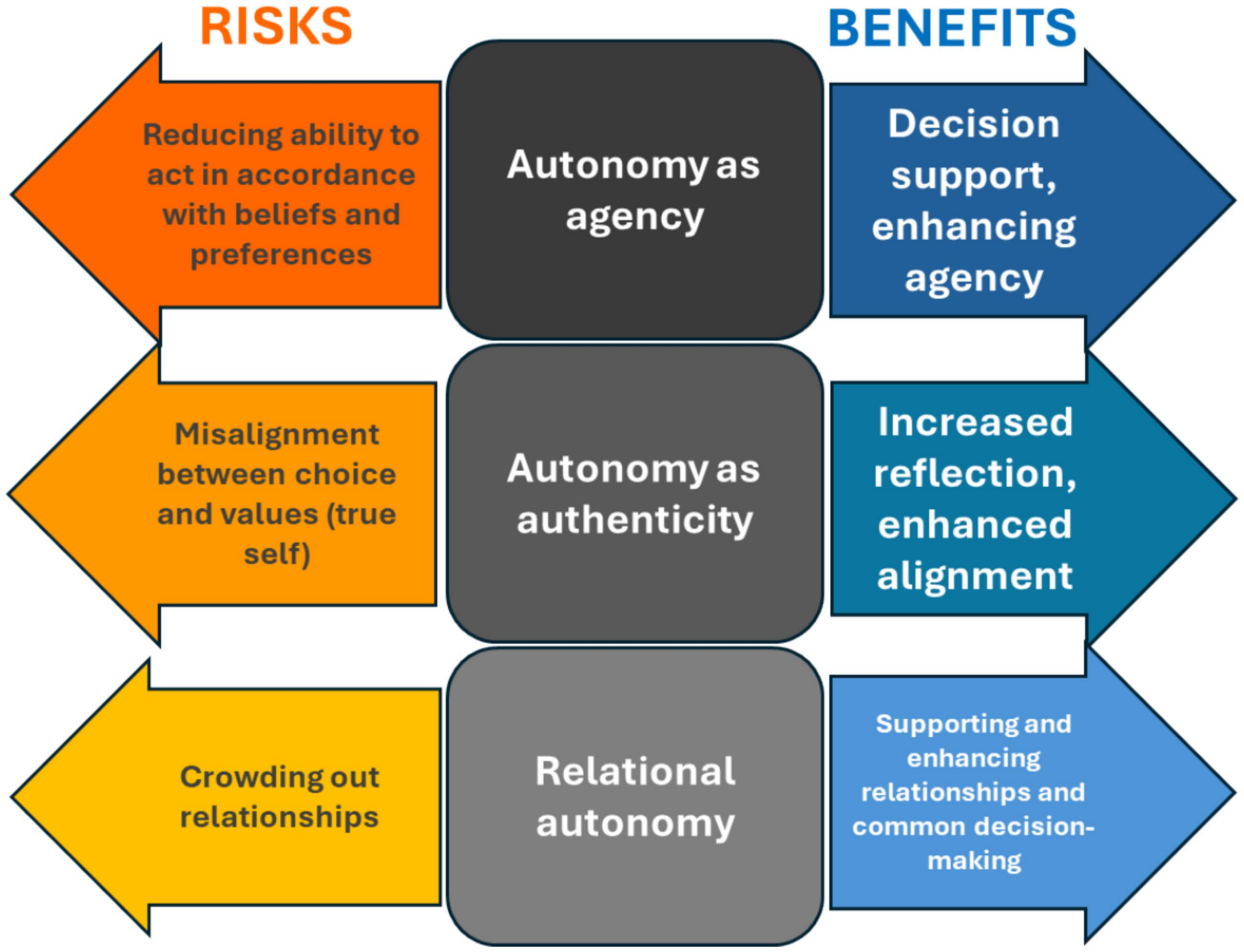
Negative implications and positive implications on three conceptions of human autonomy.

Figure 2 summarizes the implications of AI systems on three basic conceptions of human autonomy. While AI systems have the potential to enhance autonomy in its three major conceptions, it may also undermine agency, authenticity, and relational autonomy. The task then becomes to promote the first and avoid the latter. In particular, to avoid human autonomy to be overrun by AI systems, we need to pay special attention to the systems’ influence on human agency, how the structure of preferences, norms, and values is affected, and how autonomy-related relationships are swayed.

### Artificial (externalist) agency

Yet another, and partly related, way that AI systems may undermine, or challenge human autonomy is when these systems appear and act as autonomous agents.

While traditional AI systems work autonomously but follow direct human input, what has been called agentic AI systems operate independent of immediate human directions, but within limits set by humans. A third type of autonomous AI systems are often called “AI agents” and communicate and adapt to context independent of human beings, potentially in networks (Goyal, 2025; Nisa et al., 2025; Humberd and Latham, n.d.). Both traditional AI systems, AI agents, and agentic AI systems can represent a kind of “algorithmic autonomy” and are relevant for and can influence human autonomy.

In actions involving artifacts “every consequence arises from complex technological interactions, rendering these consequences difficult to predict and control by individual human agents” (Xia, 2025). Accordingly, humans lose their feeling of agency and thereby their autonomy. “[A]rtifacts have, at least partially, substituted for the lost portion of human agency and thereby causally contributed to outcomes of AIA [actions involving artifacts]” (Xia, 2025). Hence, if one applies an externalist perspective of agency, artifacts like AI systems reduce human agency over the consequences of actions as humans lose control over AI systems. Reduced agency implies reduced autonomy through reduced understanding, control, and voluntariness (Xia, 2025). By appearing as agents, AI systems gain autonomy, which can conflict with or reduce human autonomy.

In sum this analysis of “artificial autonomy” demonstrates that AI systems can undermine human autonomy by influencing all the basic constituents of autonomy, i.e., understanding, decision-making competence, and voluntariness. Moreover, AI systems can undercut autonomy in any of the three traditional conceptions of autonomy, such as *agency*, *authenticity*, and *relational autonomy*. Additionally, such systems can challenge human autonomy in terms of their apparent and efficient autonomy. Thus, “artificial autonomy” may mean two things, i.e., (1) that the autonomy humans think they have, becomes merely apparent. and (2) that AI systems acting as autonomous in making decisions for humans and transmitting goals, beliefs, preferences, and values. In both cases AI systems can come to act paternalistically. Accordingly, it is crucial to analyze this “algorithmic paternalism”.

### Algorithmic paternalism

While algorithmic paternalism has been mentioned in a wide range of publications, for example when AI systems are designed to determine and maximize persons’ and populations’ best interests (Kenig et al., 2024; McCradden and Kirsch, 2023; Kenig and Muntaner Vives, 2024; KENIG and VIVES, 2025; Meier, 2025; Salah et al., 2024; McCradden et al., 2025; Weinberger et al., 2025; Zenkl, 2025; García-Marzá and Calvo, 2024), deeper analyses are rare. In the context of medicine, it has been pointed out that “[a]lthough modern medicine has moved away from the paternalistic approach in recent decades, the advent of AI as a decisionmaking tool could potentially shift this dynamic back to the paternalistic approach, with AI presenting itself as an infallible all-knowing machine that should not be doubted” (Kenig et al., 2024). Moreover, it has been argued that “AI paternalism, or machine paternalism, is a newly described term that refers to independent decision-making by AI with no, or minimal patient participation,” (Kuhler, 2021). Correspondingly, McDougall argues that in implementing AI systems “we risk a shift back to more paternalistic medicine in a different guise” (McDougall, 2019).

More generally, it has been argued that AI systems tend to become paternalistic by ignoring the socio-cultural context of human preferences. “Ethical knowledge is not merely data about preferences—it is embedded in cultural, temporal, and subjective frames of valuation. Algorithmic paternalism—like all technocratic moral engineering—collapses these frames into monolithic categories and thereby annihilates their meaning” (Wright, 2025).

Voinea and colleagues see “paternalistic AI” as an example of a more general “technological paternalism” (Voinea et al., 2024), being as paternalism in general, defined by limiting individual users’ freedom without their consent and for their envisioned best interest (Dworkin, 2014). Correspondingly, Michael Kühler defines “AI paternalism” in the context of health apps as “capable of showing a paternalistic goal-oriented behavior toward users in that they (a) might interfere with their liberty or influence their autonomy, (b) of which users might not even be aware or at least have not consented to on specific occasions, and (c) do so because of their goal-oriented programming in terms of promoting users’ (quantifiable) health” (Kühler, 2022).

Hence, there are some conceptions of AI-related paternalism. However, we still need to investigate what kind of paternalism AI systems may provide. As indicated in Table 2, there are many kinds of paternalism, and AI systems may come to promote all of them. As Michael Kühler has assessed potential paternalism in the use of health apps, his appraisal provides a nice starting point for an analysis of algorithmic paternalism.

**Table 2:** AI interfering with different types of paternalism as categorized in Dworkin (2020).

**Weak paternalism:** AI interfering with a person’s means to contribute to the person’s ends.	**Strong paternalism:** AI interfering with people’s ends if they are considered to have mistaken or confused ends.
**Soft paternalism (Ascertaining autonomy):** AI used to ascertain understanding and voluntariness. Hindering a person to suffer harm due to ignorance.	**Hard paternalism:** AI used to interfere with people’s expressed ends, reducing their freedom of action independent of their autonomy.
**Libertarian paternalism:** Nudging AI influencing people’s choice architecture in order to help them making choices that are good for them.	**Non-libertarian paternalism:** Ignoring people’s choice architecture. Compulsion Strong paternalism Hard paternalism
**Moral paternalism:** AI influence justified by promoting the moral behavior or welfare.	**Welfare paternalism:** AI justified by promoting the (physical or mental) welfare of the person.
**Impure paternalism:** The AI system restricts or shapes the choices of others (in a more general group) in order to protect a target group.	**Pure paternalism:** The AI system restricts or guides the very people that are to be protected.

Kühler argues that AI health apps can exercise neither *weak* nor *strong paternalism*, as they cannot distinguish between health as a means or a goal (Kühler, 2022). However, AI systems have been used to predict the preferences of people in many fields, such as in healthcare (Ferrario et al., 2023; Biller-Andorno and Biller, 2019; Earp et al., 2024), entertainment (Maroto-Gómez et al., 2023), marketing, travel (Topal and Uçar, 2019), online lodging systems (Ma et al., 2021), and policy making (Lim and Savulescu, 2025). AI systems have also been able to identify human instructions, intentions, revealed preferences, and interests (Gabriel, 2020). Hence, it is reasonable to think that AI systems are able to identify means and ends, and thus to exercise both *weak* and *strong paternalism*. For example, AI systems may by various types of analyses and profiling be able to identify individuals’ preferences and provide or prescribe means according to these preferences (soft paternalism). Additionally, AI systems may override people’s goals and values (strong paternalism). Importantly, the whole alignment-debate is about whether AI systems will become paternalistic when not aligning to human values (Hristova et al., 2025; Christian, 2020).

In the context of health apps, Kühler argues that *hard paternalism* only comes into play if these “are linked to suitable environmental controls, for only then are such apps able to interfere with users’ freedom of action” (Kühler, 2022). However, in the context of AI in general, it is possible for AI systems to reduce peoples’ freedom of action, e.g., by blocking access to certain choices, and thus to exercise *hard paternalism*. The analysis of how AI systems may violate human autonomy by undermining voluntariness above provides examples of this.

When it comes to *soft paternalism*, Kühler argues that “it is hard to imagine how AI health apps would be able to check users’ (sufficient) autonomy” (Kühler, 2022). However, AI systems can provide adapted information and check and direct persons choices contingent on whether they have understood key elements of the alternative actions. Furthermore, AI systems can provide adapted information and check whether people have understood key elements before making specific decisions (Jha and Topol, 2018). In that case, AI systems could exercise *soft paternalism* as well. It may be worth noting that this goes beyond just promoting autonomy by providing more information (Kühler, 2022).

Kühler acknowledges that AI systems exercise “nudging paternalism” in health apps (Kühler, 2022). Given the increasing empirical evidence on the nudging capacity of AI systems (Beraldo, 2025; Calboli and Engelen, 2025; Fritzen, 2023), it appears clear that they may exercise *libertarian paternalism*. This has raised some concern: “At AI-driven scale, nudging threatens to turn ‘soft-paternalism’ into a kind of soft totalitarianism, to the detriment of long-term vitality of human reason. The key problem is to instead chart a course for AI agents that can help us maintain effective agency in an ever more complex world, while allowing us to maintain autonomy” (Koralus, 2025). Accordingly, AI systems may induce non-libertarian paternalism, when becoming authoritarian (Startari, 2025). As illustrated above, AI systems may nudge in a number of ways, e.g., by undermining voluntariness.

Additionally, AI systems may be advocated in terms of promoting moral behavior (moral paternalism), e.g., in framing and promoting (im)moral actions (Fossa, 2025). AI systems may also be used in “pure paternalism,” e.g., to protect a person or group from harm, e.g., when a hospital decision support system signals when a patient attempts to refuse a life-saving standard treatment. Conversely, AI systems may exhibit “impure paternalism” when restricting or shaping the choices of the general public in order to protect a target group, for example in AI-driven opioid prescribing blockade and infection control.

In sum, it may be argued that algorithmic paternalism can occur in all the traditional kinds of paternalism, i.e., strong/weak, hard/soft, and as libertarian paternalism. Table 2 provides an overview of how AI can be paternalistic. Accordingly, algorithmic paternalism may represent not only an unfair distribution of power but a dangerous combination of biased information, inappropriate recommendations, swayed judgments, and detrimental decisions. Even relatively well-designed models could drift away from person’s or groups’ true wishes, hallucinate results and obscure the reasons for their outputs, making AI systems interfere with peoples’ choice architecture and influence less trustworthy elements in decision-making.

Analysing AI in terms of traditional conceptions of paternalism can guide us in how we should identify and address paternalism in AI systems. In particular, we can investigate whether the AI system interferes with a person’s means to contribute to the person’s ends (weak paternalism), if it interferes with people’s ends if they are considered to have mistaken or confused ends (strong paternalism), whether the system is trying to avoid harm due to a person’s ignorance (soft paternalism), and if it interferes with people’s expressed ends independent of their autonomy (hard paternalism). Moreover, we can investigate whether and how a person is nudged (libertarian paternalism). Again, a detailed description of specific measures is the next step after providing this overarching framework.

As algorithmic paternalism can be analysed in traditional conceptions of paternalism, it is not new and completely different (*aliud omnino*). However, its subtlety, complexity, and obscurity make it a special challenge to human autonomy and therefore deserving special attention. What appears unique to algorithmic paternalism is that there apparently is no pater^2^ in the paternalism. There is no intentional agent in algorithmic paternalism. However, the intentionality criterion has been debated (Kühler, 2022), and it has been maintained that structural, rather than intentional, sources constitute technological paternalism (Hofmann, 2003) of which algorithmic paternalism may be a specific kind. By reducing or directing human cognition and control, AI systems may demote human autonomy and become paternalistic (Xia, 2025). Moreover, AI systems are not only able to simulate human beings beyond recognition (passing the Turing test), but are able to generate a kind of intimacy, which makes their output (advice, support, or decisions) compelling (Harari, 2024; Harari, 2018).

This relates to another important issue, i.e., the definition of “best interest” in algorithmic paternalism. How do we know that AI systems restrict people’s autonomy in those persons’ best interest, and not in the interest of others, including the AI systems’ developers or providers? This relates to the alignment problem (i.e., whether the values of the AI system align with that of the users or beneficiaries) and the black box problem (as there is no pater to ask or hold accountable). Hence, algorithmic paternalism may be more obscure (and manipulative) than human paternalism.

The main objective of this study has been to investigate how human autonomy is influenced and undermined by AI systems and how this triggers existing and special kinds of paternalism. While the analysis is conceptual and overarching, it points to some practical implications for the design, development, implementation, regulation and use of AI systems. Clearly, it is beyond the scope of this article to make a detailed evaluative framework, as this warrants a separate study. However, the analysis points to main aspects to include in an evaluative framework, shown in Table 3.

**Table 3:** Preliminary outline of an evaluative framework for assessing artificial autonomy and algorithmic paternalism.

Aspect of autonomy and paternalism	Issue to address	Phase of AI (design, development, implementation, use)	Description and strength of the problem	Detection and mitigation strategy
Understanding	Fake facts			
	Misinformation			
	Bias			
	Opacity			
	Hallucination			
	Model drift			
Competency (to make decisions)	Deskilling			
	Overreliance			
	Automation bias			
	Miscalibrated trust			
	Offloading			
Voluntariness	Soft coercion			
	Manipulation			
	Hidden intent			
	Habit-forming design			
	Norm coercion			
	Forced acceptance			
Agency	Reduced ability to act in accordance with beliefs and preferences			
Authenticity	Misalignment between choice and values (true self)			
Relational autonomy	Crowding out relationships			
Paternalism type	Reduced autonomy			
	Weak/Strong, Soft/Hard, moral, libertarian			

## Discussion

In this article I have acknowledged that AI systems have the potential to enhance human autonomy. However, closer scrutiny has revealed that such systems can undermine key requirements for human autonomy, i.e., understanding, decision-making competence, and voluntariness in a number of ways. Moreover, I have investigated how AI systems can come to breach with three standard conceptions of autonomy, i.e., autonomy as agency, as authenticity, as well as relational autonomy. Additionally, I have investigated and described how AI systems’ agentic appearance and effects can influence and threaten human autonomy. Hence, “algorithmic autonomy” may both refer to AI systems undermining the preconditions for human autonomy and the reduced human autonomy due to agentic appearance of AI systems. Correspondingly, I have argued that algorithmic paternalism can play out in all the traditional conceptions of paternalism, i.e., both as weak and strong paternalism, soft and hard paternalism, and as libertarian paternalism. Hence, there are good reasons to be aware of the paternalistic effect of AI systems, i.e., algorithmic paternalism. The main insight is that AI systems come to appear autonomous (artificial autonomy) and exhibit (algorithmic) paternalism reducing human autonomy.

The analyses have followed traditional conceptions of autonomy and paternalism, and do not lead to claims of AI exceptionalism, i.e., that AI autonomy and paternalism is exceptional. However, as the study reveals, the complexity and the obscurity of the AI systems and their effects can generate constellations of apparent autonomy and paternalism that threaten human autonomy in unprecedented ways. Accordingly, the presented analysis can be useful for identifying and explicitly addressing these issues.

There are certainly many limitations with this study. The literature on AI systems and their influence on human choice architecture is burgeoning, and it is clear that, although the reference list is long, I have not been able to address all the relevant and interesting contributions that are made. Correspondingly, many other references could have been added to Table 1, e.g., (Siau and Wang, 2020; Holdren et al., 2016; Crawford et al., 2019; Deepmind, 2025; Google, 2018; Cutler et al., 2019; Prunkl, 2024). Moreover, the searches were not systematic, and are subject to biases. However, the aim was to provide examples and arguments, and not a comprehensive and exhaustive overview of the literature. Future systematic searches and analyses are most welcome. Moreover, the analyses of the identified studies may be influenced by my conceptual perspectives on autonomy. However, the articles are identified and the reader may scrutinize my interpretations.

Moreover, there may be other conceptions of autonomy that are relevant to AI systems than those covered here, e.g., sovereignty (Sheintul, 2025). Moreover, the types of autonomy addressed here (agency, authenticity) may have other names (self-agency, autocracy) in the literature. However, I have addressed the main traditional conceptions of autonomy (Brock, 1988; Christman, 2003; Christman, 1989; Feinberg, 1982; Gómez-Vírseda et al., 2019; May, 1994; Scoccia, 1990; Young, 2017). The same goes for paternalism, where I have related to the most referred conceptions (Dworkin, 2014; Dworkin, 2015; Grill and Hanna, 2018; Scoccia, 2018). Due to the special character of AI systems, it makes sense to use the concept algorithmic paternalism.

Nonetheless, there is a socio-cultural limitation to my analysis as the frameworks for autonomy and paternalism mainly reflect Western philosophical traditions. AI, however, is implemented and used globally, and may be interpreted differently across cultural, institutional, or socio-technical contexts. This is only partly covered by relational autonomy and warrants a separate study. Moreover, the role of autonomy and paternalism is specifically important in individualistic cultures while other aspects may be more challenged in non-western societies deserving distinct attention (Yam et al., 2023).

Clearly AI influences autonomy in more complex ways than covered in this study. For example AI systems influence human voluntariness through our choice architecture, but also by our understanding, and decision-making competence, e.g., through deskilling, cognitive offloading, situational awareness, and over-reliance. Additionally, there are effects of influences on human brain, which are not addressed in this study, which has focused on the ethical aspects.

Additionally, other relevant issues and concepts, such as integrity, dignity, and privacy, have not been discussed. While relevant, they are topics for further studies.

As noted, the concept of paternalism may appear irrelevant as there is no pater. However, the paternalism framework has developed independent of identifying a “pater” and may still be relevant. Moreover, the STS literature has long been confident with artifacts having agentic effects (Xia, 2025; Aysan, 2026).

Moreover, I have not said anything about is whether the various kinds of paternalism are good or bad. This must be decided in context. For example, in a market setting, it has been argued that “Algorithms can often reach more efficient choices; they can increase one’s well-being, at least in some cases; and they do not necessarily clash with liberal political theory rationales” (Gal, 2018). The point here has been to investigate the various ways that AI systems can undermine autonomy and exercise paternalism, and by increased awareness hoping to facilitate the autonomy-enhancing applications of AI systems and avoiding autonomy-undermining uses. Correspondingly, the figures may indicate that the benefits and harms are balanced (as the arrows have the same size but opposite directions). This may of course vary greatly for various AI systems.

While this study builds on existing literature, it adds to it in four specific ways. First, it synthesizes a broad range of literature in AI, ethics, psychology, and social science. Second, it clarifies the concept of algorithmic paternalism and explicates what type of paternalism is at play. Third, it coins and defines the concept “artificial autonomy” and explains its dual nature. Fourth, the study provides an analytical framework to identify how AI systems can undermine human autonomy and exercise paternalism in order to mitigate it. However, this study is only the first step toward a detailed and practically applicable framework to address artificial autonomy and algorithmic paternalism. Further research should engage in a more detailed outline of Table 3, empirical applications to novel AI systems, and revisions and refinements.

The concerns raised in this study go beyond the fear of being outsmarted by AI systems (Nick, 2014). “Being overpowered” by paternalism is related to, but still distinct from, “being outsmarted” by superintelligence because it relates to human agency, authenticity, and relations. Hence, AI systems have a transformative and disruptive potential – they may not only change our behavior and lifeworld but also who we are ass autonomous human beings. By altering basic human characteristics, such as autonomy, which are encoded in social and legal documents, AI systems threaten to destabilize key social institutions and structures.

The implication of this study is not that we should avoid AI systems because they can undermine human autonomy or become paternalistic. It is as with other emergent and potentially disruptive technologies, that we should be philosophically proactive as reflection appears slower than technological development. Moreover, lessons from the implementation and use of social media have shown that *post hoc* reflection and regulation is ineffective (Valle et al., 2025). To ascertain that we obtain the envisioned benefits and avoid or reduce the potential harms, it is crucial that we address potential challenges up front.

## Conclusion

Acknowledging AI systems’ potential to enhance human self-determination this study has investigated how such systems can challenge human autonomy understood as agency, authenticity, and relational autonomy. By distorting understanding, decision-making competence, and voluntariness, AI systems can undermine human autonomy. As AI systems become humanlike, intimate, influence our choice architecture, predict our preferences, and impact and make our decisions they come to exercise “artificial autonomy” and enact “algorithmic paternalism”. While algorithmic paternalism can take the form of traditional conceptions of paternalism, it can combine and cloak paternalism in new and unprecedented ways. The uniqueness of algorithmic paternalism lies in its pervasiveness, complexity, and obscurity.

Like many other technologies, AI systems have a transformative and disruptive potential. Moreover, special attention is warranted as these systems may change our self-conception and autonomy. While AI systems develop fast, our reflections, norm-formations, and regulations are slow. We therefore need to be proactive in order to ascertain that we obtain the envisioned benefits and avoid or reduce the potential harms. Paying attention to and addressing the mechanisms of artificial autonomy and algorithmic paternalism is but one crucial step in preserving human autonomy.

## Data Availability

The original contributions presented in the study are included in the article/supplementary material, further inquiries can be directed to the corresponding author.

## Footnotes

1 While decision-making competence (or competency) is more often used in legal context and decision-making capacity is more often used in (bio)ethical context, I here use the first to denote the general ability to deliberate and make decisions.

2 I owe this phrase to Jonathan Adams.
